# Does Combined Physical and Cognitive Training Improve Dual-Task Balance and Gait Outcomes in Sedentary Older Adults?

**DOI:** 10.3389/fnhum.2016.00688

**Published:** 2017-01-18

**Authors:** Sarah A. Fraser, Karen Z.-H. Li, Nicolas Berryman, Laurence Desjardins-Crépeau, Maxime Lussier, Kiran Vadaga, Lora Lehr, Thien Tuong Minh Vu, Laurent Bosquet, Louis Bherer

**Affiliations:** ^1^Interdisciplinary School of Health Sciences, University of OttawaOttawa, ON, Canada; ^2^Department of Psychology, Concordia UniversityMontréal, QC, Canada; ^3^Sports Studies, Bishop’s UniversitySherbrooke, QC, Canada; ^4^Centre de Recherche de l’Institut Universitaire de Gériatrie de MontréalMontréal, QC, Canada; ^5^Department of Psychology, Université du Québec à MontréalMontréal, QC, Canada; ^6^Medecine, Université de MontréalMontréal, QC, Canada; ^7^Laboratoire MOVE (EA6314), Faculté des Sciences du Sport, Université de PoitiersPoitiers, France

**Keywords:** dual-task gait, dual-task balance, combined training (physical and cognitive dual-task training), transfer effects

## Abstract

Everyday activities like walking and talking can put an older adult at risk for a fall if they have difficulty dividing their attention between motor and cognitive tasks. Training studies have demonstrated that both cognitive and physical training regimens can improve motor and cognitive task performance. Few studies have examined the benefits of combined training (cognitive and physical) and whether or not this type of combined training would transfer to walking or balancing dual-tasks. This study examines the dual-task benefits of combined training in a sample of sedentary older adults. Seventy-two older adults (≥60 years) were randomly assigned to one of four training groups: Aerobic + Cognitive training (CT), Aerobic + Computer lessons (CL), Stretch + CT and Stretch + CL. It was expected that the Aerobic + CT group would demonstrate the largest benefits and that the active placebo control (Stretch + CL) would show the least benefits after training. Walking and standing balance were paired with an auditory n-back with two levels of difficulty (0- and 1-back). Dual-task walking and balance were assessed with: walk speed (m/s), cognitive accuracy (% correct) and several mediolateral sway measures for pre- to post-test improvements. All groups demonstrated improvements in walk speed from pre- (*M* = 1.33 m/s) to post-test (*M* = 1.42 m/s, *p* < 0.001) and in accuracy from pre- (*M* = 97.57%) to post-test (*M* = 98.57%, *p* = 0.005).They also increased their walk speed in the more difficult 1-back (*M* = 1.38 m/s) in comparison to the 0-back (*M* = 1.36 m/s, *p* < 0.001) but reduced their accuracy in the 1-back (*M* = 96.39%) in comparison to the 0-back (*M* = 99.92%, *p* < 0.001). Three out of the five mediolateral sway variables (Peak, SD, RMS) demonstrated significant reductions in sway from pre to post test (*p-values* < 0.05). With the exception of a group difference between Aerobic + CT and Stretch + CT in accuracy, there were no significant group differences after training. Results suggest that there can be dual-task benefits from training but that in this sedentary sample Aerobic + CT training was not more beneficial than other types of combined training.

## Introduction

Standing balance and walking are two motor activities that are assumed to be automatic in nature and not requiring a great degree of conscious thought. However, in real life situations, it is rare that we are simply standing still or walking without attending to some secondary task (i.e., walking and talking). Research that examines the ability to attend to two tasks simultaneously during gait and balance suggests that both younger and older adults involve some executive processes (planning, inhibiting, and switching/coordinating) in order to manage these dual-task situations (Woollacott and Shumway-Cook, 2002; Allali et al., 2008; Hausdorff et al., 2008). A large literature associates these executive processes with the prefrontal cortex (PFC; Miyake et al., 2000; Stuss and Alexander, 2000; Gunning-Dixon and Raz, 2003). The PFC has been shown to be an area that is sensitive to age-associated declines with aging (Buckner, 2004; Hedden and Gabrieli, 2004). Moreover, studies suggest that cognitive functions largely subserved by the PFC can be improved through physical exercise and cognitive training interventions (Colcombe and Kramer, 2003; Erickson et al., 2007; Kramer and Erickson, 2007; Braver et al., 2009; Klingberg, 2010; Bherer et al., 2013). Given the potential plasticity of the PFC and its association with executive functions, the goal of the current study was to assess the benefits of combined physical and cognitive training on dual-task gait and balance in a sample of sedentary older adults.

Although both younger and older adults may involve executive function processes to manage dual-task gait and balance, the literature suggests that the ability of older adults to manage such situations is reduced in comparison to younger adults (Woollacott and Shumway-Cook, 2002; Fraser et al., 2007; Li et al., 2012; Fraser and Bherer, 2013). Further, when active and sedentary older adults are compared, sedentary older adults demonstrate a greater risk of cognitive and physical declines that can influence executive functions and ultimately increase fall risk (Thibaud et al., 2011). This risk increases in dual-task situations (Montero-Odasso et al., 2012). In addition, certain types of dual-task combinations can increase the dual-task interference (Fraser and Bherer, 2013). In particular cognitive tasks that are more executive in nature seem to interfere more with gait (Al-Yahya et al., 2011; Buracchio et al., 2011; Walshe et al., 2015). A systematic review and meta-analysis found that cognitive tasks with “mental tracking” (pg. 725) increased the interference during walking to a greater degree than simple reaction time tasks (Al-Yahya et al., 2011). The literature on motor-cognitive dual-tasks has certainly demonstrated that both cognitive and motor factors might be contributing to these age differences in dual-task abilities (Woollacott and Shumway-Cook, 2002; Hausdorff et al., 2008; Schaefer and Schumacher, 2010). In general, older adults with mild cognitive impairment or dementia tend to walk more slowly than healthy older adults (Montero-Odasso et al., 2012). In addition, the measurement of gait during dual-task has also revealed differences in gait parameters between healthy and cognitively impaired seniors (Sheridan et al., 2003; Springer et al., 2006; Muir et al., 2012) and fallers and non-fallers (Springer et al., 2006; Montero-Odasso et al., 2012). Such findings suggest that dual-task gait might be an important clinical marker of cognitive decline and falls risk (Montero-Odasso et al., 2012).

Similarly, maintaining standing balance is considered a complex skill that requires several elements and cognitive processing has been identified as one of them (Horak, 2006). In comparison to the gait literature, the literature on dual-task balance assessments demonstrates that this approach has been successful in differentiating fallers from non-fallers (Brauer et al., 2001; Condron et al., 2002; Hauer et al., 2003) and somewhat successful in differentiating healthy older adults from those with cognitive impairment (Zijlstra et al., 2008; Muir-Hunter et al., 2014). More research using static balance dual-tasks is required to determine if this type of assessment can identify different forms of cognitive impairment. Despite this, gait and balance are both postural control tasks and common to both is the association with executive functions (van Iersel et al., 2008) and the potential for cognitive plasticity.

Cognitive plasticity and enhanced motor function have been targeted with intervention studies. Several intervention studies have demonstrated that exercise training can improve cognitive outcomes (Predovan et al., 2012; Bherer et al., 2013; Langlois et al., 2013; Berryman et al., 2014; Bherer, 2015) and physical outcomes (Shumway-Cook et al., 1997; Cadore et al., 2013; de Labra et al., 2015). Regarding specific types of exercise training, aerobic training in particular has been associated with enhanced executive functions (Kramer et al., 1999; Hall et al., 2001; Colcombe and Kramer, 2003) and increased activations in the frontal and parietal cortices (Colcombe et al., 2004, 2006). However, most review and meta-analyses tend to suggest that exercise intervention combining aerobic and strength training components lead to greater improvement in cognition (Colcombe and Kramer, 2003). Cognitive training (CT) can also lead to improvement (Segev-Jacubovski et al., 2011; Belleville and Bherer, 2012) in several cognitive domains including working memory, attention, and executive functions (Li et al., 2008; Kueider et al., 2012; Leung et al., 2015). There is however, some debate about the transfer of cognitive training to untrained tasks or everyday activities (Lee et al., 2012; Lussier et al., 2015). Despite this, some cognitive training interventions have shown transfer demonstrating improved motor outcomes in older adults in balance (Li et al., 2010), gait (Verghese et al., 2010), balance and gait (Smith-Ray et al., 2013) and activities of daily living (Willis et al., 2006). More specifically, in the first three studies mentioned, participants received a computerized cognitive training (seated at a computer) and outcome measures such as mediolateral sway (Li et al., 2010), gait speed in single and dual-task walking (Verghese et al., 2010) and timed-up-and go and distracted walking (Smith-Ray et al., 2013) improved in comparison to control groups that did not receive this training.

When exploring training specific to motor-cognitive dual-task situations, a systematic review specific to dual-task outcomes, revealed that motor-cognitive dual-task training was beneficial to standing balance performance and that walking outcomes were improved by both single-task and dual-task training (Wollesen and Voelcker-Rehage, 2013). It is important to note that a majority of the studies contained in the review trained the component tasks either separately (cognitive task alone or motor task alone) or concurrently (dual-task condition in which cognitive and motor task were trained simultaneously), then assessed single- and dual-task performances with the same combination of tasks. None of the studies looked specifically at combined cognitive and physical training and transfer to untrained dual-task balance and gait tasks.

A recent systematic review of combined cognitive and physical training interventions in older adults with or without cognitive impairment demonstrated that this type of combined training was beneficial in two of the three studies on older adults without cognitive impairment and four out of the five studies examining individuals with cognitive impairment (Law et al., 2014). Only one of the studies contained in the review examined dual-task walking as an outcome measure and included an active placebo control group that received low intensity exercise training (Schwenk et al., 2010). This study found that training older individuals with cognitive impairment in dual-task walking, at an adaptive level of difficulty (challenging the patients), led to improvements in dual-task walk outcomes in comparison to the placebo control. While this study is explicitly highlighted as a well-designed randomized control trial in the review, there were a few critiques. Specifically, components of the dual-task assessed at pre-and post-intervention were similar to the tasks being trained and the placebo control did not have the same hours of training.

Taken together, there are few studies that have explicitly examined transfer from a combined cognitive and physical training program to dual-task gait and balance outcomes in a sample of sedentary older adults. There were several outcome measures included in this large mobility study (see Desjardins-Crépeau et al., 2016). Only the results specific to balance and gait dual-task outcomes are reported here. The goal of the current study was to assess whether or not combined cognitive and physical training benefits transferred to dual-task walk and balance outcomes and whether or not there would be synergistic effects when combining cognitive dual-task training and aerobic training which have demonstrated specific benefits to executive function. In the present study, there were four training groups including either aerobic, cognitive or active control conditions. The first three groups all contained components designed to improve executive function and the last group was considered our active placebo-placebo control. All groups were expected to benefit to some degree from training but we expected the Aerobic + CT group to demonstrate greater improvements in dual-task walking and balance outcomes than the other three groups after training. Further, we expected that the placebo-placebo control would demonstrate the least amount of benefits to dual-task walking and balance outcomes in comparison to the three other groups which all included conditions designed to improve executive function.

## Materials and Methods

### Participants

Sedentary older adults (≥60 years old) who performed less than 150 min of physical activity per week were recruited from public advertisements (flyers, newspapers) and from the research center’s participant pool. One hundred and thirty six individuals passed our phone screening, which had the following exclusion criteria: history of neurological disease or major surgery in the year preceding the study, uncorrected auditory or visual impairments, smoking, severe mobility limitations or any other physical activity contraindications, and being currently engaged in any structured physical activity. The 136 were randomly assigned to one of the four training groups (Aerobic + Cognitive Training (CT); Aerobic + Computer lessons (CL); Stretch + CT, Stretch + CL). Our Aerobic + CT group contained both physical and cognitive components to improve executive function and our placebo-placebo control containing no aerobic or cognitive dual-task training was the Stretch + CL group. Of those 136, 11 abandoned the study before beginning any training (for personal reasons, time constraints, or recent injuries that restricted their ability to participate). Of the 125 who entered into the study and provided written and informed consent that conformed to the Montreal Geriatric Institute ethics committee and the Declaration of Helsinki, 22 abandoned during training and 31 were excluded, at post-test, for the following reasons: wearing a hearing aid (affected performance on auditory task with headset), having a Geriatric Depression Score greater than 11 (which is suggestive of mild/moderate depression), and insufficient training (less than 75% of training sessions completed). Reasons for abandoning typically related to: injury outside the lab that impeded participation; death in the family; and other commitments. Those who abandoned were equally distributed across the training groups. This study was carried out in accordance with the recommendations of human research by the ethics committee of the Montreal Geriatric Institute. The protocol was approved by this ethics committee.

Descriptive characteristics (i.e., age, education, etc.) of the participants included in the final sample (*n* = 72; 51 women/21 men) are presented in Table 1 at the beginning of the “Results” Section. It is important to note that we began balance assessments in the third cohort recruited for this study, as such, there are fewer participants in each of the training groups that completed the dual-task balance assessments. For dual-task balance, *n* = 15 (Aerobic + CT); *n* = 12 (Aerobic + CL); *n* = 16 (Stretch + CT); and *n* = 11 (Stretch + CL).

**Table 1:** **Descriptive characteristics of the participants by training group**.

	Aerobic + CT	Aerobic + CL	Stretch + CT	Stretch + CL	Group differences? *p*-value
Participants	21	17	18	16	
Age (years)	71.90 (6.84)	70.53 (7.34)	72.22 (5.93)	71.13 (5.40)	0.86
Education (years)	13.81 (2.62)	16.06 (2.19)	13.89 (4.39)	15.00 (2.73)	0.11
6MWT (meters)	508.45 (75.49)	538.53 (76.39)	511.50 (80.81)	513.06 (62.68)	0.61
PPT (score)	32.40 (2.42)	32.18 (3.52)	31.83 (1.92)	33.00 (1.83)	0.59
ABC (score)	82.68 (2.9)	83.82 (2.9)	85.03 (2.9)	89.08 (3.2)	0.49
MMSE	28.48 (1.25)	28.88 (1.05)	29.39 (0.70)	28.50 (1.37)	0.06
GDS	4.57 (3.34)	4.18 (2.68)	3.11 (3.31)	2.44 (2.37)	0.14

*Note: 6MWT, 6 min walk test (total meters completed in 6 mins); PPT, modified Physical Performance Test (max score 36); ABC balance questionnaire (max score 100% confidence in balance abilities); MMSE, Mini Mental State Exam (a global measure of cognition, max score 30); GDS, Geriatric Depression Scale (scores equal to or above 11 are indicative of mild/moderate depression)*.

### Protocol

All participants underwent pre-testing across 3 days. On Day 1, pre-testing involved: a comprehensive medical exam with a geriatrician. On Day 2, participants completed a full neuropsychological battery and a dual-task cognitive training baseline on the computer (see Desjardins-Crépeau et al., 2016 for a detailed description of the protocol). Finally on Day 3, participants completed a battery of physical tests (including the 6 min walk test, the timed-up-and-go, and the short physical performance battery) and dual-task walking and balance assessments. Upon completion of the pre-testing groups of 4–8 individuals began their respective training protocol, which involved 12 weeks of training 3 times a week. All participants had two 60 min sessions of physical exercise (aerobic or stretch) and one 60 min session of cognitive stimulation (dual-task training or computer lessons). The mixed aerobic training involved a 5 min warm up, 15 min of lower body resistance training, 30 min of cardiovascular exercise on a treadmill and a cool down. Intensity of the exercise increased over the sessions based on each individuals’ ratings of perceived exertion on the Borg (1998) scale. The Stretching and toning group also had a 5 min warm-up and cool down but spent the majority of the training time (50 min) performing whole body stretching exercises mainly in a seated position. The computer dual-task training involved two visual discrimination tasks (number and shape discrimination) that were based on previous cognitive training paradigms (Bherer et al., 2005, 2006, 2008). Participants were encouraged to be as accurate and rapid as possible at responding to the tasks alone (single-task) and simultaneously (dual-task). Participants were provided continuous feedback on their response time (during the session) and provided feedback for both response time and accuracy at the end of each session. In contrast to the dual-task training, those who received computer lessons had demonstrations and trials with different computer applications (i.e., word and excel) and learned how to search the internet. At the end of the 12 weeks, all participants returned for 2 days of post-testing. The first day of post-testing involved completing the same neuropsychological assessments completed at pre-test and the second day of post-testing involved completing the same battery of physical tests and dual-task walk and balance assessments.

### Main Outcome Measures

#### Cognitive Accuracy: N-back Task (With and Without Concurrent Walking/Balance)

The n-back task is a working memory task that can be parametrically manipulated to increase the memory load during testing (Jaeggi et al., 2003; Doumas et al., 2008). Typically during an n-back task, a series of stimuli are presented and the participant is asked to remember and respond to the stimuli that they heard “n” items-back (0-back, 1-back, etc.). In the lowest load version (0-back), individuals simply have to remember and report the stimuli they just heard. As the number of items back increases, the working memory load increases placing greater attentional demands on the individual (Jaeggi et al., 2003; Doumas et al., 2008). The auditory n-back used in this study involves working memory and mental tracking as the participant has to remember the numbers that are continuously being presented and as a new number is presented say out-loud the number they heard one item back. During the balance task, in order to minimize muscle fatigue from repeated balance assessments (Helbostad et al., 2010), we chose to complete the dual-task balance with the 1-back only. During the walk portion, we used two levels of difficulty the 0-back and the 1-back version of the task (with and without concurrent walking). In all conditions (single (n-back only) and dual-task (n-back + motor task)), a mean accuracy score was computed (percent correct of total possible responses (%)).

**Figure 1:**
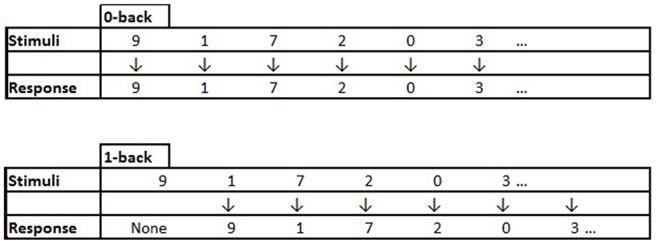
**An example of the stimuli presented and vocal response expected from the participant for the 0-back and 1-back conditions.** *Note*: In the 0-back condition the participant hears a number “9” and immediately responds “9”. In the 1-back condition the participant hears the first stimuli “9” and has to keep it in mind until they hear the second stimuli “1” and at this point they respond with the first item they heard “9”, then they have to keep in mind the “1” until presented with the next stimuli, and so on.

The n-back task in the current study has already successfully demonstrated differences in cognitive performances during walking in sample of older women who completed a combined training intervention (Fraser et al., 2014). In this auditory n-back, the to-be-remembered stimuli were numbers (0–9). A visual depiction of the 0-back and 1-back conditions is presented in Figure 1. The numbers were recorded in a female voice and soundfiles (wavfiles) of each number were used in pseudo-randomly ordered lists of numbers that were presented using E-prime2 software (Psychology Software Tools Inc., Sharpsburg, PA, USA). The numbers were pseudo-randomly ordered to ensure that there were no repeats (9-9) and no ordered series (1-2-3). The numbers were presented through wireless headphones (Sennheiser Canada, Pointe-Claire, QC, Canada). For DT-balance, six lists of eight numbers were created; two were used for practice and the remaining for the test phase. For DT-walk, 10 lists of 12 numbers were created; two lists were used for practice and the remaining for the test phase. Since there were two levels of n-back in the DT-walk these lists were used twice.

#### Mobility Measures: Posture and Gait

In both balance and the walk portions of the physical assessment after instructions were provided, the participants wore headphones for both the practice and test phases. Prior to any testing, the dominant leg of the participant was assessed by having the participant begin walking from a standing position three times (Fraser et al., 2007). The leg most often used for gait initiation was considered the dominant leg. For the balance portion participants had to maintain balance with their eyes open, arms at their sides, on their dominant leg for 20 s with and without the 1-back task. Postural control was assessed with a Matscan floor mat that captures the plantar pressures and forces of the foot (Tekscan, Boston, MA, USA). The Matscan raw data was converted to center of pressure measures. In the current study, we measured area (cm^2^), velocity, peak to peak dispersion, and standard deviation of sway values specific to the mediolateral (ML) and anterior-posterior (AP) directions. Based on the training benefits reported in ML sway in the study by Li et al. (2010), we have focused on area and ML sway variables measured during single- and dual-task balance (Peak ML (cm), SD ML (cm), RMS ML (cm^2^), Velocity ML (cm/s)). Mean scores for each of the ML sway values were computed. In addition, at the end of the pre- and post-test sessions, we asked each participant to complete the Activities-Specific Balance Confidence (ABC) scale (Powell and Myers, 1995) in order to have a standardized questionnaire of balance confidence in everyday activities. The scale has 16 items which are rated on a 0%–100% scale of confidence, higher scores (closer to 100%) indicate that the individual is completely confident in their balance ability during the activity in question. A mean confidence value from all 16-items was calculated for Pre- and Post-test.

For the walk portion, participants had to walk down a 37-m hallway at a comfortable self-selected pace for 30 s with and without the n-back task. Each meter in the hallway was marked on the floor and an experimenter remained with the participant during walk trial and recorded the number of steps taken. The participant was cued to stop and remain still at the end of each trial by a beep in the head set and the experimenter measured from the dominant heel to the nearest meter marked on the floor to obtain accurate measures of distance. Each walk trial had a fixed time (30 s), therefore meters per second (walk speed) was calculated for each participant. Mean walk speed values (m/s) were calculated for each participant for single and dual-task walking.

#### Procedure: Pre-Test and Post-Test Dual-Task Assessments

Each participant included in the final sample completed pre- and post-training physical assessments. At the beginning of each physical assessment session, participants were weighed (kg) and their height (cm) and abdominal circumference (cm) measurements recorded. In a seated position the participants were explained the n-back task and told that they would complete this task while seated and while balancing on one foot. They then completed single-task cognitive (SC; 1-back only) practice. Once their dominant leg was determined, the participant then practiced single balance (SB; balancing only) and practiced dual-task balancing (DTBal; balance and 1-back). All trials SC, SB, DT-bal were 20 s in duration and started with three warning beeps and finished with a single beep. After practice, the participants completed the test phase, in which the condition order was (ABCCBA): SC, SB, DTBal, DTBal, SB, SC. This ABCCBA order allowed the different conditions to be distributed throughout the test phase and fatigue in any one condition minimized.

Once the balance portion of the physical assessment was complete, the experimenter explained the walk portion of the experiment. Similar to the balance portion the condition order for the walk portion had practice on each condition (SC, single walk (SW), and DT-walk) followed by a similar ABCCBA order to the balance test. All conditions were 30 s in duration and began with three beeps and finished with one beep. In comparison to the balance portion, the walk portion had additional conditions such that each participant had the following test order (SC, SC, SW, DT-walk, DT-walk, DT-walk, DT-walk, SW, SC, SC). Each participant completed this order with the 0-back task and then with the 1-back task.

### Statistical Analyses

#### Training Effects

Prior to presenting any transfer from training to our single and dual-task walk outcomes, it is important to state whether or not the four groups improved after training. With respect to physical training, we expected groups who received aerobic training would have greater physical benefits than our groups who received stretch training. Improvements in physical training were assessed with Pre-Post 6 min walk test (6MWT). A one way ANOVA on mean post-test scores for this variable was conducted to assess group differences. With respects to cognitive dual-task computer training, we examined if groups who received cognitive training were able to diminish their dual-task costs on a visual dual-task to a greater extent to those who did not receive cognitive dual-training. Change scores were computed ((dual-task costs (PRE)-dual-task costs (POST))/dual-task costs (POST)). Groups with higher change scores demonstrate diminished dual-task costs after training. These change scores were also subjected to a one way ANOVA to test group differences in training effects.

#### Single and Dual-Task Walk Data

A full model was conducted to test within and between main effects and interactions for our variable of interest (walk speed). In order to assess difficulty effects, tasks effects, time effects and training group differences in walk speed, we conducted a 2 × 2 × 2 × 4 ANOVA on mean walk speed with the following within-subjects factors: Task (Single vs. Dual), Time (Pre vs. Post-test), and Difficulty (0-back vs. 1-back) and the between-subjects factor was Group (Aerobic + CT, Aerobic + CL, Stretch + CT, Stretch + CL). The same ANOVA was used to test any mean differences in accuracy (% correct).

#### Single and Dual-Task Balance Data

The analysis of the dual-task balance data differed from the walk data in that it involved only one level of the n-back task (1-back). For each ML-sway variable and cognitive accuracy score (means only), 2 × 2 × 4 ANOVAs were conducted with the within-subjects factors of Time (Pre vs. Post), Task (single vs. dual) and the between-subjects factor of Group (Aerobic + CT, Aerobic + CL, Stretch + CT, Stretch + CL). For all statistical analyses, IBM SPSS Statistical package version 21 was used, the alpha was set at (0.05) for significance and all *post hoc* comparisons were Bonferroni corrected.

## Results

### Training Effects

The one way ANOVAs on physical improvements after training revealed that all our groups improved on their 6MWT and there were no significant differences between the groups (*p* = 0.21). The ANOVA on the dual-task cost change scores revealed significant differences between the groups, *F*_(1,67)_ = 4.63, *p* = 0.005, *η*^2^ = 0.17. The groups that received cognitive training had higher change scores than those that did not receive cognitive training and there were no differences in the two groups that received cognitive training (Aerobic + CT and Stretch + CT; *p* = 0.22). Stretch + CT dual-task cost change (*M* = 1.19) was greater than Aerobic + CL (*M* = 0.02; *p* = 0.002) and Stretch + CL (*M* = 0.05; *p* = 0.005). Aerobic + CT dual-task cost change (*M* = 0.74) was greater than Aerobic + CL (*M* = 0.02; *p* = 0.04) and marginally greater than Stretch + CL (*M* = 0.05; *p* = 0.07).

### Single and Dual-Task Walk Data

The walk speed means were subjected to a 2 × 2 × 2 × 4 ANOVA in order to assess within-subjects effects of difficulty, task and time and between-subjects effects of group. The ANOVA revealed a main effect of time, *F*_(1,68)_ = 27.83, *p* < 0.001, *η*^2^ = 0.29, in which walk speed means were greater at post-test (*M* = 1.42 m/s, *SE* = 0.03 m/s) than at pre-test (*M* = 1.33 m/s, *SE* = 0.03 m/s). The ANOVA also revealed a main effect of task, *F*_(1,68)_ = 193.92, *p* < 0.001, *η*^2^ = 0.74, in which participants walk speeds were greater in single-task (*M* = 1.39 m/s, *SE* = 0.02 m/s) than in dual-task (*M* = 1.35 m/s, *SE* = 0.02 m/s). With respects to difficulty, there was also a main effect, *F*_(1,68)_ = 37.80, *p* < 0.001, *η*^2^ = 0.36, in which participants walked faster in the more difficult 1-back condition (*M* = 1.38 m/s, *SE* = 0.02 m/s) than in the 0-back condition (*M* = 1.36 m/s, *SE* = 0.02 m/s).

**Figure 2:**
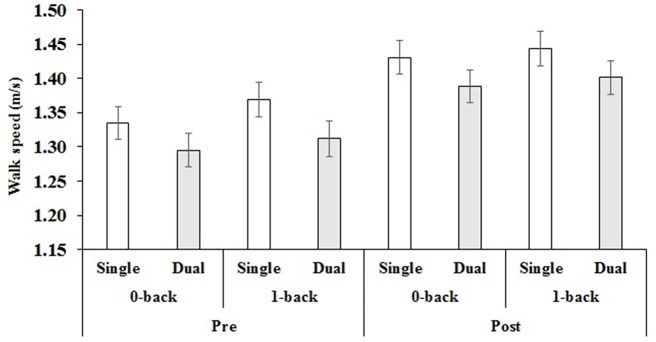
**Mean pre- and post-test values for single and dual-task walk speed for both difficulty levels.** *Note*: Error bars = standard error of the mean.

The ANOVA also revealed two significant interactions. The first, a task by difficulty interaction *F*_(1,68)_ = 4.56, *p* = 0.036, *η*^2^ = 0.06, supported the main effect findings such that single-task (ST) walk speed was greater than dual-task (DT) walk speed in both 0-back (*M*_ST_ = 1.38 m/s, *SE*_ST_ = 0.02 m/s > *M*_DT_ = 1.34 m/s, *SE*_DT_ = 0.02 m/s) and 1-back (*M*_ST_ = 1.41 m/s, *SE*_ST_ = 0.02 m/s > *M*_DT_ = 1.36 m/s, *SE*_DT_ = 0.02 m/s). Further, although in both difficulty levels there were significant differences in ST and DT, the mean difference was higher in the 1-back contrast (0.05 m/s) than in the 0-back contrast (0.04 m/s). In addition, a time by task by difficulty interaction, *F*_(1,68)_ = 5.83, *p* = 0.02, *η*^2^ = 0.08, demonstrated that there were differences between single-task and dual-task at each difficulty level, such that walk speed was greater in single-task vs. dual-task and greater in 1-back vs. 0-back, but that these differences were less at post-test (*p-values* for contrasts < 0.02) compared to pre-test (*p-values* for contrasts <0.003; see Figure 2 for mean single and dual-task values across time and difficulty level). Please see Table 2 for the mean dual-task walk speed values across time, difficulty level and group.

**Table 2:** **Dual task mean values for walk speed and accuracy for each group, time and difficulty level**.

	Aerobic + CT				Aerobic + CL			
Time	Pre		Post		Pre		Post	
Difficulty	0-back	1-back	0-back	1-back	0-back	1-back	0-back	1-back
**Walk speed (m/s)**	1.27 (0.05)	1.28 (0.05)	1.36 (0.04)	1.37 (0.05)	1.28 (0.05)	1.31 (0.05)	1.42 (0.05)	1.43 (0.05)
**Accuracy (%)**	99.89 (0.30)	91.74 (1.47)	100 (0.18)	94.16 (1.24)	99.33 (0.33)	97.59 (1.64)	100 (0.20)	96.52 (1.38)

	**Stretch + CT**				**Stretch + CL**			
**Time**	**Pre**		**Post**		**Pre**		**Post**	
**Difficulty**	**0-back**	**1-back**	**0-back**	**1-back**	**0-back**	**1-back**	**0-back**	**1-back**

**Walk speed (m/s)**	1.27 (0.05)	1.29 (0.05)	1.39 (0.05)	1.39 (0.05)	1.36 (0.05)	1.37 (0.06)	1.38 (0.05)	1.41 (0.05)
**Accuracy (%)**	100 (0.32)	96.59 (1.59)	99.62 (0.19)	96.97 (1.34)	100 (0.06)	93.18 (1.69)	100 (0.20)	97.87 (1.42)

*Note: Standard error of the mean (SE) reported in the brackets beside the mean value*.

### Single and Dual-Task Accuracy Data (% Correct)

**Figure 3:**
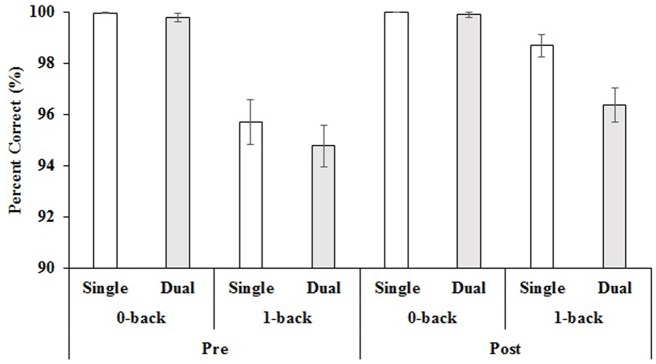
**Mean pre- and post-test values for single and dual-task accuracy for both difficulty levels.** *Note*: Error bars = standard error of the mean.

Similar to the walk speed data, the mean accuracy scores (% correct) were subjected to a 2 × 2 × 2 × 4 ANOVA in order to assess within-subjects effects of difficulty, task, and time and between-subjects effects of group. There were four significant main effects and three interactions with difficulty. There was a main effect of time, *F*_(1,68)_ = 8.38, *p* = 0.005, *η*^2^ = 0.11, in which accuracy was higher at post-test (*M* = 98.57%, *SE* = 0.20%) than at pre-test (*M* = 97.57%, *SE* = 0.36%). There was also a main effect of task, *F*_(1,68)_ = 7.80, *p* = 0.007, *η*^2^ = 0.10, in which accuracy was higher in single-task (*M* = 98.60%, *SE* = 0.25%) than in dual-task (*M* = 97.72%, *SE* = 0.27%). The main effect of difficulty, *F*_(1,68)_ = 72.64, *p* < 0.001, *η*^2^ = 0.52, went in the expected direction with participants providing more accurate responses in the easier 0-back (*M* = 99.92%, *SE* = 0.05%) in comparison to the more difficult 1-back condition (*M* = 96.39%, *SE* = 0.41%). There was also a main effect of group, *F*_(1,68)_ = 2.96, *p* = 0.038, *η*^2^ = 0.12, in which the Aerobic + CT group had lower accuracy scores (*M* = 97.18%, *SE* = 0.38%) than the three other groups (Aerobic + CL (*M* = 98.29%, *SE* = 0.42%); Stretch + CT (*M* = 98.70%, *SE* = 0.41%); and Stretch + CL (*M* = 98.46%, *SE* = 0.44%)). This group effect was further qualified by a significant difficulty by group interaction, *F*_(1,68)_ = 3.24, *p* = 0.027, *η*^2^ = 0.13, in which the Aerobic + CT group had lower 1-back accuracy scores (*M* = 94.38%, *SE* = 0.76%) than the Stretch + CT group (*M* = 97.50%, *SE* = 0.82%) only. In addition to this interaction, there was a difficulty by time interaction, *F*_(1,68)_ = 7.08, *p* = 0.01, *η*^2^ = 0.09, which demonstrated that there were no differences pre to post-test (*p* = 0.49) in the 0-back condition, but there were significant improvements from pre (*M* = 95.25%, *SE* = 0.71%) to post-test (*M* = 97.54%, *SE* = 0.41%) in the 1-back condition. Finally, there was a difficulty by task interaction, *F*_(1,68)_ = 6.27, *p* = 0.015, *η*^2^ = 0.08, in which there were only significant differences in single (*M* = 97.21%, *SE* = 0.50%) and dual-task (*M* = 95.58%, *SE* = 0.53%) in the 1-back condition. Please see Table 2 for the mean dual-task accuracy values across time, difficulty level and group. Please see Figure 3 for mean single- and dual-task accuracy across time and difficulty level.

### Single and Dual-Task Mediolateral Balance Data

The balance dual-task involved only one level of difficulty, the 1-back. We conducted 2 × 2 × 4 mixed ANOVAs (time by task by group) on each sway variable and mean accuracy (% correct). For velocity there were no significant effects. For Peak sway, there was a main effect of time *F*_(1,50)_ = 3.99, *p* = 0.05, *η*^2^ = 0.07, in which Peak sway was higher at Pre-test (*M* = 11.68 cm, *SE* = 1.02 cm/s) than at Post-test (*M* = 9.80 cm, *SE* = 0.95 cm). The standard deviation (SD) sway variable also demonstrated a significant main effect of time *F*_(1,50)_ = 5.73, *p* = 0.02, *η*^2^ = 0.10, in which SD sway was higher at Pre-test (*M* = 2.31 cm, *SE* = 0.22 cm) than at Post-test (*M* = 1.84 cm, *SE* = 0.21 cm). A significant time by task by group interaction *F*_(1,50)_ = 3.40, *p* = 0.025, *η*^2^ = 0.17 was also found for the SD variable. *Post hoc* analyses revealed that the Stretch + CL group had a significant difference (*p* = 0.001) in SD single-task (balancing alone) from Pre (*M* = 2.86, *SE* = 0.49) to Post (*M* = 1.42, *SE* = 0.48) while all other groups did not demonstrate any significant differences. The root mean squared (RMS) variable also demonstrated a main effect of time *F*_(1,50)_ = 4.26, *p* = 0.04, *η*^2^ = 0.08, in which RMS was higher at Pre-test (*M* = 25.40 cm^2^, *SE* = 0.89 cm^2^) than at Post-test (*M* = 23.47 cm^2^, *SE* = 0.92 cm^2^). In addition, there was a significant time by task interaction *F*_(1,50)_ = 7.55, *p* = 0.008, *η*^2^ = 0.13, in which single-task RMS was not significantly different from Pre- to Post-test (*p* = 0.14) but that dual-task RMS was higher at Pre-test (*M* = 25.50 cm^2^, *SE* = 0.90 cm^2^) than at Post-test (*M* = 23.04 cm^2^, *SE* = 0.91 cm^2^; *p* = 0.015). For the 1-back mean accuracy during balancing, the 2 × 2 × 4 mixed ANOVA revealed a main effect of time *F*_(1,50)_ = 16.61, *p* < 0.001, *η*^2^ = 0.24, in which Post-test accuracy was higher (*M* = 95.97%, *SE* = 0.75%) than Pre-test accuracy (*M* = 91.72%, *SE* = 1.09%). There was also a main effect of task *F*_(1,50)_ = 4.36, *p* = 0.042, *η*^2^ = 0.08, in which the single-task 1-back accuracy (*M* = 95.10%, *SE* = 0.89%) was higher than the dual-task 1-back accuracy (*M* = 92.59%, *SE* = 1.07%). There were no other significant effects or interactions. Table 3 presents the mean dual-task mediolateral sway values for each group pre- and post-test.

**Table 3:** **Mean dual-task mediolateral (ML) sway values for each group and time**.

	Aerobic + CT		Aerobic + CL		Stretch + CT		Stretch + CL	
Time	Pre	Post	Pre	Post	Pre	Post	Pre	Post
**Velocity (cm/s)**	4.87 (0.70)	3.69 (0.56)	4.40 (0.79)	3.47 (0.63)	5.07 (0.68)	5.13 (0.54)	4.22 (0.82)	4.05 (0.65)
**Peak (cm)**	11.72 (2.16)	8.83 (1.68)	11.28 (2.41)	6.96 (1.88)	12.64 (2.09)	10.92 (1.63)	10.27 (2.52)	11.36 (1.96)
**SD (cm)**	2.24 (0.47)	1.64 (0.47)	2.13 (0.52)	1.45 (0.53)	2.40 (0.45)	2.16 (0.46)	2.12 (0.55)	2.37 (0.55)
**RMS (cm^2^)**	23.57 (1.68)	21. 94 (1.71)	26.78 (1.88)	22.27 (1.92)	24.76 (1.63)	23.32 (1.66)	26.88 (1.97)	24.64 (2.00)
**Accuracy (%)**	87.16 (3.43)	97.14 (1.81)	93.39 (3.56)	98.98 (1.87)	87.14 (3.43)	92.86 (1.81)	90.08 (3.68)	93.97 (1.94)

*Note: Standard error of the mean (SE) reported in the brackets beside the mean value*.

## Discussion

In the current study, age-associated changes in physical and cognitive function were targeted with a combined training (physical and cognitive) protocol. Participants were randomly assigned to one of four different training protocols (Aerobic + CT, Aerobic + CL, Stretch + CT, Stretch + CL). The goal of the study was to assess whether or not the benefits of a combined training protocol would transfer to untrained gait and balance dual-tasks. Based on the literature, it was hypothesized that the Aerobic + CT group would demonstrate the greatest improvements in dual-task gait and balance in comparison to other groups. In addition, we predicted that the Stretch + CL group, our active control, would demonstrate the least improvements in dual-task gait and balance after training. Our findings only partially support our hypotheses, as we did find improvements in dual-task walk and balance outcomes from pre to post-test, but we did not find that our Aerobic + CT group improved to a greater degree than the other groups, or that our placebo-placebo control (Stretch + CL) improved the least.

### Training Effects

All groups demonstrated improvements in our outcome measures for physical (6MWT) and cognitive dual-task training (dual-task cost change scores). In terms of physical training, the 6MWT did not reveal any differences between our groups in performance gains, such that the groups containing aerobic training did not benefit to a greater degree than the groups containing stretch training. For the cognitive training, a majority of the group contrasts suggest that the cognitive dual-task training was beneficial in reducing dual-task costs post-test for the groups that received this specific type of training compared to the other groups who had computer lessons.

### Single and Dual-Task Walk Findings

Consistent across all groups, and confirming the dual-task effect, walk speed and accuracy were higher in single-task conditions vs. dual-task conditions. Interestingly, when examining main effects of difficulty, all groups walked faster in the harder difficulty level (1-back) when compared to the easier difficulty level (0-back) but all were less accurate in the 1-back compared to the 0-back. Increasing the speed of the motor response to dual-task conditions is similar to the facilitation findings we have found in dual-task treadmill walking (Fraser et al., 2007). When the walk speed was fixed, participants responded more rapidly to the cognitive task during walking when compared to responding in a seated position. Perhaps the ability to speed up ones response cognitively or in the present study increase walk speed in a more challenging dual-task allows for better management of the walking dual-task. The task by difficulty effect in walk speed does support that the 1-back difficulty level was more difficult than the 0-back difficulty level as the difference between single and dual-task walk speed were greatest in the 1-back condition. Further, accuracy levels overall were high, but 1-back accuracy levels are clearly poorer than 0-back accuracy levels which also support the manipulation of difficulty.

The full model also revealed that all the participants in our sample benefited from training. Post-test walk speed and accuracy values were greater than pre-test values and the differences between single and dual-task walk speeds were diminished at post-test. With the exception of a slight accuracy difference between our Aerobic + CT group and our Stretch + CT group, our walk results do not reveal specific group differences but rather demonstrate that all groups improved in their accuracy and walk speed from pre to post-test. Despite the lack of significant differences found in the full statistical model, from a clinical standpoint 0.05 m/s increase in walk speed represents a small meaningful change and 0.10 m/s represents substantial meaningful change (Perera et al., 2006; Kwon et al., 2009). In the current study, the only group that did not demonstrate a clinically significant change in walk speed from pre to post-test was our active placebo control. Groups with an aerobic and/or cognitive training component demonstrated 0.09 m/s increase in walk speed or greater. As such, from a clinical point of view, it may be more beneficial to have an aerobic and/or a cognitive training component in a combined training protocol.

The results of the full model differ from the study by Schwenk et al. (2010) that targeted dual-task walk speed as a primary outcome and found significant group differences in their most difficult dual-task gait condition.

There are several possible reasons for these contrasting findings. First, Schwenk et al. (2010) chose a type of exercise training that was very close to their outcome measure, as such they successfully demonstrated near transfer (training effects in a task similar to the trained task). Our training protocol did not explicitly train dual-task walking abilities but rather focused on aerobic exercise and cognitive dual-task training at a computer. As such, our training was distinct from our outcome measure and we were assessing far transfer effects to dual-task walking. Our results suggest that any type of active intervention in a sample of sedentary older adults can improve dual-task gait. In addition, while both studies included a 12-week training protocol, in Schwenk et al.’s (2010) study the groups did not have the same number of intervention hours (control group had half the time of the experimental group), whereas all participants in our 12-week intervention were exposed to the same number of intervention hours and always trained in groups. It is unknown how this influences the differences in our findings but it is possible that the differences between our groups were reduced as they all had the same number of intervention hours. Another important difference between the studies, is that Schwenk et al. (2010) trained older adults with cognitive impairment (dementia) and we trained individuals without cognitive impairment. A meta-analysis examining the effects of aerobic exercise on cognitive performance suggests that greater improvements in memory may be seen in those with cognitive impairment compared to those without cognitive impairment (Smith et al., 2010). Although sedentary, our sample was relatively healthy, did not have cognitive impairment or functional limitations and this may have minimized the gains seen after training. One interesting similarity between the two studies is that the most consistent findings emerge in the more difficult condition. In our study, although we found robust differences in v and 1-back performances supporting a difficulty effect, it is possible that if we challenged our groups with an additional difficulty level (for example a 2-back) we would see differences between our training groups on a more challenging dual-task walk situation. Also, in the current study, we chose to focus on walk speed, in a relatively high functioning sample, other gait parameters (e.g., stride time variability) might have been more sensitive to training induced changes (Lamoth et al., 2011.

While it was important in the current study to control for the number of visits and interactions with training groups by including active control groups, another possible reason for improvements across all the groups may have been our alternate choice in physical training (stretch training). Although the stretch training in the current study did not target aerobic capacity it did have a resistance training component and the goal of improving lower body strength. The improvement in lower body strength may have transferred to improvements in walk speed. Indeed, there is some evidence that certain types of stretching/resistance training exercises can influence gait speed (Stanziano et al., 2009; van Abbema et al., 2015).

### Single and Dual-Task Balance Findings

The main findings from the mediolateral sway measures were that all participants improved from pre to post-test in Peak sway, SD, RMS. In the SD variable, there was a time by task by group interaction in which the active placebo-placebo (Stretch + CL) had a significant improvement in single-task performance from pre-to post and the other groups did not demonstrate this effect. This finding should be interpreted with caution, as direct comparisons of the groups on the single-task sway measures by means of a one-way ANOVA did not find any significant differences between the groups. The RMS sway value also demonstrated a task by time interaction in which there were no improvements pre to post in single-task performances but there were improvements in dual-task performances (reduced sway). It is important to note also that our standardized paper and pencil measure of balance confidence, the ABC scale did not reveal any differences between our groups and all groups had high scores suggesting that our findings are not influenced by a lack of confidence in balance abilities.

In terms of accuracy on the 1-back task, all groups demonstrated the typical task effect in which single-task cognitive performance (seated in a chair) was more accurate than dual-task cognitive performance (while balancing). In addition, all groups improved their accuracy significantly from pre- to post-test. Similar to the dual-task gait findings, all participants improved in several (but not all) mediolateral postural sway variables and in their cognitive accuracy during balance. Our results also suggest that sedentary adults can demonstrate reduced sway (improved balance) after an active intervention protocol. There were limited task effects in the balance data, one interaction effect with time, task, and group in which there was single-task SD sway improvement in the Stretch + CL group that wasn’t apparent in the other groups. This finding is difficult to explain, as the groups did not differ in single-task SD when the one-way ANOVA comparing groups was conducted. It may be the case that the limited sample size in the balance data influenced the outcome of the larger 2 × 2 × 4 mixed ANOVA. The RMS variable seems to be sensitive to task effects demonstrating dual-task balance improvements over time across groups, but additional research with larger sample sizes is needed to truly evaluate the importance of these variables for outcome measures of dual-task balance.

### Limitations

Limitations specific to our training protocols and active control groups are discussed elsewhere (see Desjardins-Crépeau et al., 2016). Specific limitations for this portion of the study relate to the small sample size in the balance outcome measures, limiting our potential interpretation and the generalizability of results. Nonetheless, there is a very limited literature on dual-task static balance as an outcome measure for combined cognitive and physical training programs that do not train the components of the outcome measure being assessed (i.e., single and dual-task balance training). The results of this study can be used to inform selection of variables of interest in future combined intervention studies with static sway variables as outcomes. Regarding the dual-task gait outcomes, the sample size is larger and the changes in dual-task 1-back walk speed (see Table 1) argue against simple test re-test effects, as the three groups that had cognitive and/or aerobic training have higher gains in walk speed pre-post (Aerobic + CT: 0.09 m/s, Aerobic + CL:.12 m/s, and Stretch + CT: 0.10 m/s) than the placebo-placebo group (Stretch + CT: 0.04 m/s). Despite this, it is unclear given the findings what specifically led to improved dual-task gait performances. Given that we solicited sedentary older adults, who may have fewer outings and less social contact, it is possible that the social interaction produced by the regular group meetings contributed to improved outcomes in the current study and this not a factor that was measured in this study. It would be important in future combined cognitive and physical training studies to control for social factors of regular group sessions.

Further, it is important to note that our methodological choice of having a combined training protocol limited the number of training sessions we were able to provide to our participants on a weekly basis. Although our group has demonstrated cognitive improvements from a 12-week training protocol (Predovan et al., 2012) and balance benefits from cognitive training (Li et al., 2010) these training protocols were able to devote all the weekly sessions to a specific training type (either aerobic or cognitive) and as such might have boosted the benefits of training. In our protocol, we had to reduce the amount of cognitive training to once per week and physical training to twice per week in order to provide both cognitive and physical training in the same protocol. Wollesen and Voelcker-Rehage (2013), indicated in their review that not only did the type of training influence dual-task outcomes but the amount of training could also influence outcomes. As such, the combined protocol might have reduced the training effects as the amount of cognitive and physical training per week might not have been sufficient to demonstrate the potential synergistic effects of combined training.

## Conclusion

Given the clinical importance of dual-task gait and balance assessments in identifying individuals at risk for falls and cognitive decline and the potential for training to improve dual-task balance and gait, future combined training studies should include dual-task outcome measures in order to tease out what kind of training (perhaps adaptive), how often, at what intensity, would be most beneficial to improve dual-task balance and gait. Future studies, utilizing portable imaging technologies such as functional near infra-red spectroscopy will complement the behavioral findings of this study and provide more clarity on cognitive plasticity in the PFC. Certainly, the training gains seen across all groups in walk and certain balance measures in the current study suggest that encouraging sedentary older adults to actively participate in a training protocol may have transferrable benefits to dual-task gait and balance that could potentially reduce fall risk and cognitive decline in this population.

## Author Contributions

SAF: design, protocol, training of graduate students, data analysis, manuscript write-up. KZ-HL: design, principal investigator, secured funding for the project, trained graduate student for balance data analysis, provided feedback. NB: design for physical assessments (6MWT, etc.) training of all trainers for the physical portion of study and data analysis of the physical data. LD-C: responsible for all the testing, data entry, and analysis of the neuropsychological data. ML: responsible for the cognitive training and computer lesson component, and all the data related to this component. KV: responsible for converting the raw balance data to center of pressure scores, provided feedback on preliminary version of manuscript. LL: responsible for the physcial training with participants, data entry for physical and neuropsychological data. TTMV: medical doctor responsible for all the clinical assessments of participants, provided feedback on preliminary version of manuscript. LBosquet: design, physical assessment, one of the principle investigators who secured funding for the project. LBherer: design, cognitive training component, principle investigator who secured funding for the project—provided feedback on several versions of the manuscript.

## Conflict of Interest Statement

The authors declare that the research was conducted in the absence of any commercial or financial relationships that could be construed as a potential conflict of interest.
